# Content and Delivery of Physical Therapy in Multiple Sclerosis across Europe: A Survey

**DOI:** 10.3390/ijerph17030886

**Published:** 2020-01-31

**Authors:** Kamila Řasová, Jenny Freeman, Davide Cattaneo, Johanna Jonsdottir, Ilse Baert, Tori Smedal, Anders Romberg, Peter Feys, Jose Alves-Guerreiro, Mario Habek, Thomas Henze, Carme Santoyo-Medina, Antonie Beiske, Paul Van Asch, Daphne Bakalidou, Yeliz Salcı, Erieta Dimitrova, Markéta Pavlíková, Ivana Štětkářová, Jana Vorlíčková, Patricia Martinková

**Affiliations:** 1 Department of Rehabilitation, Third Faculty of Medicine, Charles University, Ruská 87, 108 00 Prague, Czech Republic; marketa@ucw.cz; 2 Faculty of Health: Medicine, Dentistry and Human Sciences, University of Plymouth, Devon PL6 8BH, UK; jenny.freeman@plymouth.ac.uk; 3 IRCCS Fondazione Don Carlo Gnocchi, Larice Lab, P20148 Milan, Italy; dcattaneo@dongnocchi.it (D.C.); jjonsdottir@dongnocchi.it (J.J.); 4 Faculty of Rehabilitation Sciences REVAL Rehabilitation Research Center REVAL, BIOMED, Hasselt University, 3500 Hasselt, Belgium; ilse.baert@uhasselt.be; 5 Norwegian Multiple Sclerosis Competence Centre, Department of Neurology, and Department of Physiotherapy, Haukeland University Hospital, 5021 Bergen, Norway; tori.smedal@helse-bergen.no; 6 Physiotherapy, Masku Neurological Rehabilitation Centre, 21250 Masku, Finland; anders.romberg@neuroliitto.fi; 7 Campus Diepenbeek, Faculty of Rehabilitation Sciences, REVAL Rehabilitation Research center REVAL, BIOMED, Hasselt University, 3500 Hasselt, Belgium; peter.feys@uhasselt.be; 8 School of Health Sciences, Health Research Unit, Polytechnic Institute of Leiria, Campus 2-Morro do Lena-Alto do Vieiro, 2411-901 Leiria, Portugal; jose.guerreiro@ipleiria.pt; 9 Department of Neurology, Referral Center for Autonomic Nervous System, University Hospital Center Zagreb, School of Medicine, University of Zagreb, Kispaticeva, 10000 Zagreb, Croatia; mario.habek@mef.hr; 10 Specialist Practice in Neurology, 93059 Regensburg, Germany; thomas.henze@outlook.com; 11 Neurology-Neuroimmunology Department & Neurorehabilitation Unit, Multiple Sclerosis Centre of Catalonia (Cemcat), Vall d’Hebron University Hospital, 08035 Barcelona, Spain; csantoyo@cem-cat.org; 12 Nevrologgruppen Oslo, 0159 Oslo, Norway; agbeiske@gmail.com; 13 Fit Up, Fitness- and Physiotherapy Center, 2550 Kontich, Belgium; paulvanasch@yahoo.com; 14 Department of physiotherapy, University of West Attica, Egaleo, 12243 Athens, Greece; dafbak@otenet.gr; 15 Department of Physical Therapy and Rehabilitation, Faculty of Health Science, Hacettepe University, 06100 Ankara, Turkey; fztyeliz@hotmail.com; 16 Department for Rehabilitation of Musculoskeletal Disorders, Institute of Physical Medicine and Rehabilitation, Faculty of Medicine, “Ss. Cyril and Methodius” University, 1000 Skopje, Macedonia; erietand@yahoo.com; 17 Department of Neurology, Third Faculty of Medicine, Charles University, 100 34 Prague, Czech Republic; ivana.stetkarova@fnkv.cz; 18 Department of Statistical Modelling, Institute of Computer Science, Czech Academy of Sciences,18207 Prague, Czech Republic; jane.vorlickova@gmail.com (J.V.); martinkova@cs.cas.cz (P.M.)

**Keywords:** multiple sclerosis, physical therapy, Europe, questionnaire survey, professional guidelines

## Abstract

*Background:* Guidelines and general recommendations are available for multiple sclerosis rehabilitation, but no specific guidance exists for physical therapists. Describing aspects of physical therapy content and delivery in multiple sclerosis and its determinants and analysing whether general recommendations connected with physical therapy are implemented in practice is important for interpreting clinical and research evidence. Methods: An online cross-sectional survey of physical therapists specialized in multiple sclerosis (212 specialists from 26 European countries) was used. *Results*: There was distinct diversity in service delivery and content across Europe. Perceived accessibility of physical therapy varied from most accessible in the Western region, and least in the Southern region. Sixty-four physical therapists adjusted their approach according to different disability levels, less so in the Eastern region. Duration, frequency and dose of sessions differed between regions, being highest in Southern and Western regions. “Hands on treatment” was the most commonly used therapeutic approach in all apart from the Northern regions, where “word instruction” (providing advice and information) prevailed. Conclusions: The content and delivery of physical therapy differs across Europe. Recommendations concerning access to treatment and adjustment according to disability do not appear to be widely implemented in clinical practice.

## Key Messages:

**1.** **Implications for Policy Makers**

No specific policy guidance exists for physical therapists treating multiple sclerosis.

There is a diversity in service delivery and content across Europe.

Duration, frequency and dose of sessions differ between European regions.

Accessibility of physical therapy varies between European regions.

**2.** **Implications for Public**

The article describes aspects of physical therapy content and delivery in multiple sclerosis and its determinants. It uncovered variation across European regions in the frequency of using physical therapy (PT) interventions, characteristics of therapy sessions, therapy modification and long term monitoring for people with multiple sclerosis (MS). This knowledge may help support the development of guidelines for practitioners to improve the consistency and quality of PT delivery, which aim to enhance functioning and quality of life for people with MS.

## Main Manuscript:

## 1. Background

Text for this section. Multiple sclerosis is progressive autoimmune disease that causes numerous acute or sub-acute neurological symptoms and considerable disabilities which negatively limit participation and quality of life. As a consequence, MS continues to be a disabling neurological disease that affects people at the peak of their career and causes substantial health and social care burden. Therefore, comprehensive management including rehabilitation is urgently needed to manage the wide range of complex and interacting symptoms that people are confronted with in their daily lives [1].

Due to disease modifying drugs, relapse remitting MS has a better disease course [2]; which has the potential benefit of enabling greater possibilities for rehabilitation. Our clinical experience has highlighted that a more stable disease course enables patients to engage more actively and intensively in the rehabilitation process, with more positive outcomes. There is also increasing evidence to demonstrate that rehabilitation can improve functional abilities and coping skills and improve quality of life in people with progressive MS [3], even in those with significant disability [4], as most people with MS have a normal life expectancy.

Although it has been recommended that people with MS should attend regular, long-term rehabilitation [1,5], our previous research confirmed regional disparities in:

(1) Organizational aspects of physical therapy such as teamwork, rules of PT prescriptions, specialization of centers and types of services provided [6], and
(2) The wide variety of PT interventions used by different European centers [7].

Although Europe is clearly determined from a geographical perspective, no universal European identity exists due to language, religious, philosophical and historical differences. Despite this, Europe has undergone a process of integration at a political, legislative and economic level. In line with this, experts from different European countries co-operate on important issues such as the improvement of health care. In line with this, the necessity for developing standards for MS rehabilitation services across Europe was introduced in 2008 [8]. The first recommendations for MS rehabilitation were published in 2012 and were devoted to basic organizational aspects. These included suggested requirements for: a comprehensive approach, setting short- and long-term goals from a patient-centered point of view, delivery of appropriate interventions to manage symptoms, disabilities and handicap (participation restrictions), its monitoring and adjustment, and access to inpatient, outpatient and community services [9]. Although specific recommendations for PT exist for several diseases [10], these are still waiting for MS.

We are aware that there are many different PT interventions available to treat patients with MS [7]. In addition, views about what PT entails differ. Some therapists emphasize the role of stimuli application in PT, using mechanical force and movements, manual therapy, exercise therapy and electrotherapy. Other therapists emphasize PT as a problem-solving educational process [11]. Different views could influence both the delivery and outcome of therapy. This survey intended to investigate whether some of the recommendations relevant to PT are implemented in clinical practice, namely: frequency of therapy, whether it is adjusted to disability level, and its availability of access. Information about session characteristics, long term monitoring of therapy and the respondents’ view regarding therapeutic effect were also explored.

## 2. Methods

### 2.1. Research Design

A descriptive, cross-sectional survey between individual physical therapists who work with MS patients using convenience sampling was used in this study.

### 2.2. The Survey Questionnaire

This questionnaire survey was part of the COPHYREQUEST project [6]. In previous studies, the views from country and centre representatives on MS organisation were published [12], and PT interventions used in MS patients were described [7]. In this article, answers from individual PTs focussing on content and delivery of PT interventions in MS are analysed.

We were interested in:

- whether and how individual PTs adjusted their approach according to different levels of disability,
- whether (and if so, how) they were in contact with patients after the treatment ends,
- details about their typical therapeutic sessions (length, dose and composition),
- the therapeutic approach (how much time during their therapeutic sessions was devoted to using hands-on techniques/therapeutic handling, verbal instruction/advice and information and demonstration),
- the PTs perception of treatment accessibility, effectiveness and its sustainability.

PTs were also asked to rate on an ordinal scale between 0 and 10 how accessible they feel that PT is for patients with MS in their country (10 points signifying that PT is accessible for everybody who needs it).

### 2.3. Recruitment Process

The databases of the Rehabilitation in Multiple Sclerosis Network (RIMS), European Multiple Sclerosis Platform, European Society of Physical and Rehabilitation Medicine, World Federation for NeuroRehabilitation and professional networks LinkedIn and ResearchGate were searched to identify key individuals working in the field of MS in 45 European countries. Of these, representatives from 28 countries agreed to assist both in identifying centres where people with MS undergo PT and to participate in completing the survey (6). 420 PTs from these workplaces were identified for potential participation, of whom 323 were able to answer the questionnaire in English. Of these, 133 PTs from 43 centers completed the questionnaire. An additional 79 PTs from 73 centers were recruited at conferences organized by the Rehabilitation in Multiple Sclerosis Network (www.eurims.org), also see [7].

### 2.4. Data Analyses

European regions (West, South, North, East) were defined according to the United Nations Statistics Department [13]. 26 participating countries were divided into four regions: (1) West: Austria, Belgium, France, Germany, Netherlands, Switzerland; (2) South: Croatia, Former Yugoslav Republic of Macedonia, Greece, Italy, Portugal, Serbia, Slovenia, Spain, Turkey; (3) North: Denmark, Estonia, Finland, Ireland, Norway, Sweden, United Kingdom; and (4) East: Czech Republic, Poland, Romania, Slovakia.

Differences between regions in categorical variables were tested using the Pearson Chi-squared test with a simulated *p*-value [14], due to very small counts in some cells. In case of high counts in cells (the results are similar to those using classical Chi-squared test), we used the simulated *p*-value for consistency. In the case of continuous variables, we did not assume normality of distributions (e.g., length of a therapy), and the significance of differences between regions, or between groups of PTs with different characteristics (gender, education, etc.) was tested using the Kruskal–Wallis test.

To account for multiple comparisons, Benjamini–Hochberg correction [15] was applied. Results were reported as statistically significant if the corrected *p* value was lower than 0.05. Statistical environment R, version 3.5.0, [16] and its libraries were used throughout the analyses.

## 3. Results

### 3.1. Respondents

A total of 212 respondents from 115 workplaces across 26 European countries participated in the survey (response rate 53%).

Most respondents were PTs (95%), female (73%), aged between 31 and 50 years (58%), with more than 10 years of practice (57%). In terms of qualifications, 41% had a bachelor’s, 32.5% a master’s and 8.5% a doctoral degree, 10.8% were diploma specialists and 7.1% had “other” education. Almost half worked specifically with MS patients for less than a quarter of their working time (41%). Respondents’ characteristics differed somewhat by regions (Table 1). Half of the respondents were from small centers, which provided PT for up to 100 patients a year, and over two thirds came from centers which had a small ratio (up to 20%) of patients with MS.

### 3.2. PT Interventions

**Figure 1:**
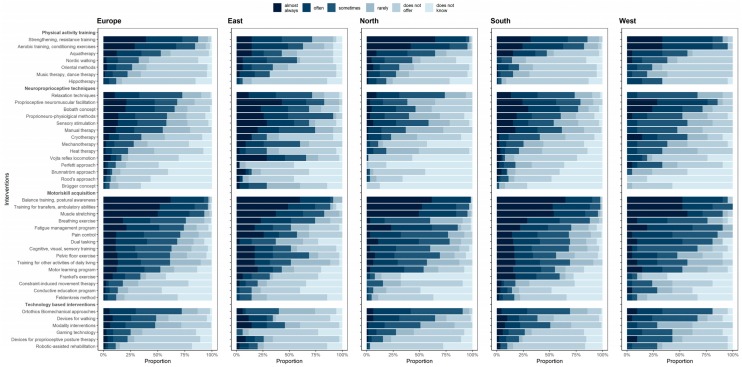
Knowledge and use of interventions by physiotherapists in Europe and four European regions.

PTs reported using a variety of interventions. Balance training and postural awareness, training for transfers and ambulatory abilities, muscle stretching, strengthening, and aerobic training are well-known interventions and the most frequently used (Figure 1). The previous publication by Martinková et al. [7] focused on region differences and other factors that matter in the use of PT interventions, finding significant region differences in most interventions. For example, Vojta reflex locomotion or the Perfetti approach are considered key interventions in one region but are used very rarely/not known at all by PTs in other regions.

Legend: Interventions are categorized according to Martinkova et al., 2018 [7], and are ordered by frequency of use across Europe (higher scores of “sometimes” or more often are near the top).

### 3.3. Perceived Accessibility of PT for People with MS

**Figure 2:**
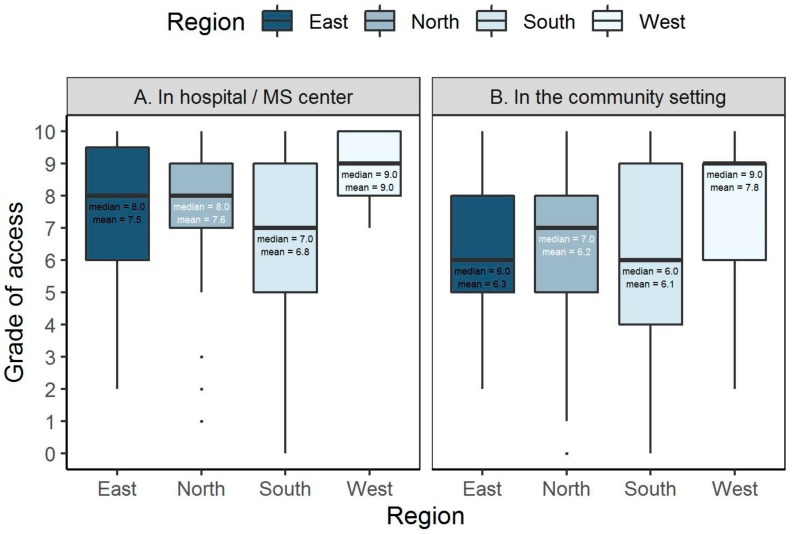
Perceived accessibility of multiple sclerosis (MS) physiotherapy by region A. In hospital/MS center. B. In the community setting. 10: Available to anyone who needs it, 0: Not available to anyone who needs it.

Regions differed in the perceived level of accessibility of PT, both for hospital and MS centers (*p* = 0.002) and the community setting (*p* = 0.042) (Figure 2). Generally, PT was reported as being more accessible in the West region (median grade 9/10) for all settings. This contrasted with other regions as follows: East region (median grade 8/10 for hospital and 6/10 for community), North (8/10, 7/10 respectively), South (7/10, 6/10 respectively).

### 3.4. Characteristics of Therapy Sessions

**Figure 3:**
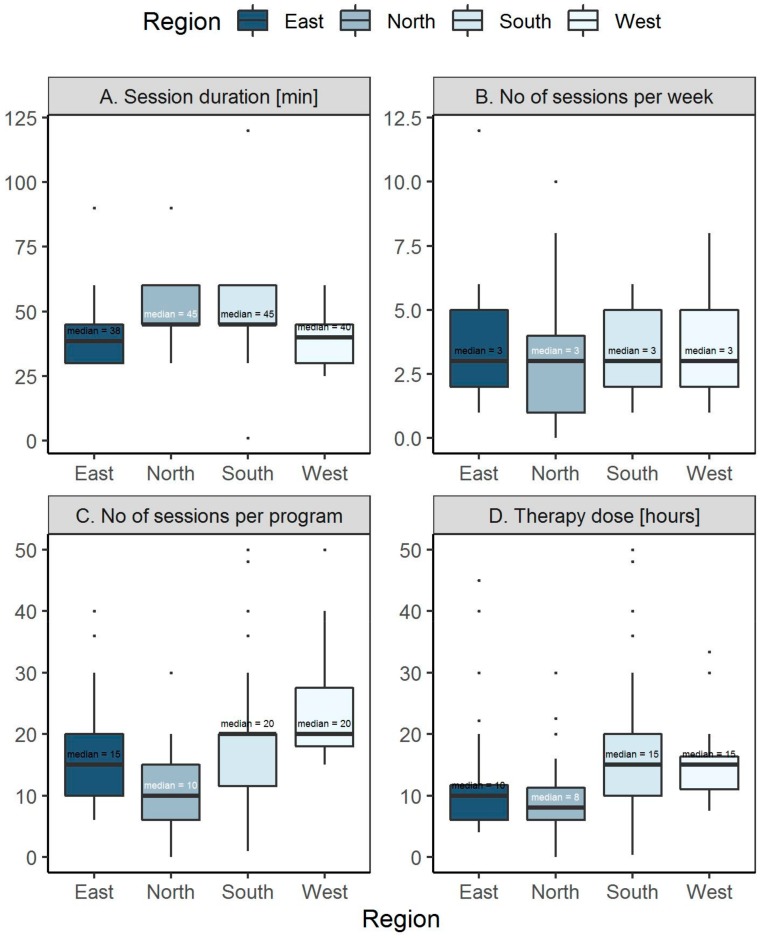
Characteristics of therapy session by regions. (**A**). Session duration. (**B**). Number of sessions per week. (**C**). Number of sessions per program. (**D**). Therapy dose (hours).

The therapy session differs between regions in all aspects studied, namely: duration (*p* < 0.001), number of sessions per program (*p* < 0.001) and dose (*p* < 0.001). In general, South and West regions provide a higher dose/longer duration of therapy input (median 15 hours’ total) than East and North regions (median 10 and 9 h total respectively) (Figure 3).

**Figure 4:**
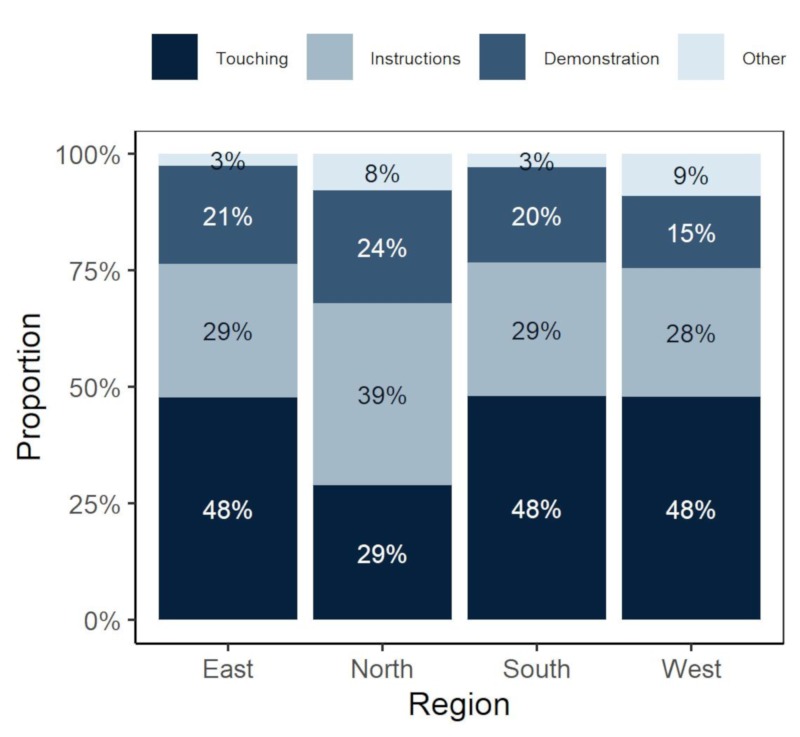
Therapy session composition by regions.

On average across all regions, therapists spend 42% of the session using hands-on techniques, 31% of the time giving instructions/providing advice and information and 22% using demonstration. PTs from the North region use hands-on techniques significantly less (*p* = 0.002) and spend more of the therapy sessions providing advice and information (*p* = 0.013) (Figure 4) than other regions.

Legend: Respondents were asked to specify how much of the time they use hands-on therapy techniques during a standard treatment (Touching) and how much of the time they use words (Instructions), demonstration (Demonstration) and other ways of leading the patient (Other). Answer should have totalled to 100%.

### 3.5. Adjusting Therapy to Level of Disability

On average, 65% of PTs reported that they adjust their therapeutic approach to different levels of disability; regions differ in this aspect (*p* = 0.009), with the East region using this approach less often (40%).

### 3.6. Long Term Monitoring

Forty-three percent of PTs reported being in contact with their patients after the treatment ends. These are mostly “outpatient visits” (61% of those who are in contact), with 9% using telerehabilitation, and 30% other forms of contact (mostly e-mail or phone calls). There was no difference in regions in this aspect.

### 3.7. Perception of PT Effect

PT was generally viewed as effective by the PTs (median 5 on a scale of 0–5, mean 4.4, SD 0.8, IQR 1) in all European regions. A third (33%) of therapists estimated that the positive effects of a face-to-face episode of inpatient//outpatient based PT intervention would persist for up to three months, with a further third (31%) estimating these effects typically last between three and six months. A minority (8%) estimated the effects would last for more than six months. The Western region was more sceptical in this estimation than other regions (*p* = 0.011).

## 4. Discussion

The processes of care and interventions used in rehabilitation are not described sufficiently in the literature [17], although the number of papers devoted to PT in MS has increased recently [1,5]. Authors [18,19,20] have highlighted the importance of paying attention to opening the black box of PT, but only a few studies have been conducted in a limited number of neurological conditions, such as stroke [21], spinal cord injury [22] and Parkinson’s disease [23], using different methodologies. To our knowledge, this is the first survey in which specialists described a variety of aspects of PT delivery in MS. We can only evaluate whether our results correspond with a recent, multi-center trial that brings real-world evidence from specialized centers [24].

Respondents from a relatively high number of countries (*n* = 26) participated in the survey, although information was unavailable from some European countries. Moreover, the number of participants in each region was not balanced. Nevertheless, the results provide a useful overview of the situation across Europe. In the interpretation of survey results, it is necessary to consider that the characteristics of respondents differed between regions. In the majority of European regions, respondents were mainly women, while in Western countries the gender ratio was more balanced. Although almost half (41%) of the responding PTs had a bachelor’s degree, there was a visible trend towards higher levels of education. Whilst PT education used to be predominantly at diploma level, nowadays professional and research doctorates and master’s degree programs are an integral part of universities [25]. The education of respondents varied significantly from country to country—in the East region, the PTs educational level was usually higher. On the other hand, PTs in the North and West regions were more frequently specialized in MS, and MS specific post-graduate training courses are commonly organized [26]. Most PTs in this survey worked at the small centers, which provide PT for up to 100 patients a year.

The answers of the individual PTs who completed this survey concurred with answers of center representatives in terms of the most frequently used PT interventions (balance and postural awareness) [7] but were in contrast with data from Kalron and colleagues regarding self-stretching [24]. The previous publication by Martinková et al. [7] highlighted significant region differences in the use of PT interventions but also found other factors that matter. We postulate that the interventions used may further correlate with other aspects of content and delivery of physical therapy in MS (length, accessibility, etc.).

Our results demonstrate that the average therapeutic session differs in duration, number of sessions and dose across regions. One positive interpretation is that PT is part of a multidisciplinary rehabilitation package which is usually tailored to suit an individual’s specific needs and therefore varies in content and intensity [1,21]. On the other hand, it would appear that some centers do not provide a sufficient volume of therapy. Kalron et al. [24] confirmed that more intense therapy is more effective [24,27]. They found that people with mild MS who had improved in the 12-item Multiple Sclerosis Walking Scale, received roughly twice the number of individual therapy sessions and twice as long compared to those who did not improve. Participants in the improved group, classified as moderately-severely disabled, received approximately 25% more individual therapy compared to those in the non-improved group. It is important that findings such as these should inform the organization of PT service delivery to optimize successful therapeutic outcomes.

Although the European Code of Good Practice in MS recommends equal rights and access to treatment, therapies and services in the management of MS [9], the results of this survey show marked disparities in the accessibility of services across geographical regions. Respondents perceived relatively poor access to PT for people with MS. This is in accordance with our previous findings [28] where access to outpatient therapy varied from 34% to 41.3% and inpatient therapy from 17.4% to 28.5% [29]. Kobelt et al. [29] highlighted PT as the most used paramedical intervention, while its frequency of use is relatively low (32.7%). The MS in the 21st Century initiative [30] set out to foster engagement through a series of open-forum joint workshops and concluded that access to appropriate care is critical. PwMS have alerted lack of access to treatment, treatment support, a reluctance on the part of some professionals to prescribe particular therapies, and lack of awareness of specific therapies/latest therapeutic options among some professionals [30]. Marziniak et al., 2018 [31] described other reasons as to why many patients only infrequently access health care services or are unable to access them easily. These included mobility restrictions, travel costs, consultation and treatment time constraints, and a lack of locally available MS expert services. Our results show that the highest perceived access to PT for MS patients is in the West region, followed by the North and East regions, with poorest access in the South. Our previous finding based on data from the centre representatives [6] alerted the potential role of financial coverage on the offer and length of PT intervention. Usual range of the coverage is 70%–100%, but some centres reported very low coverage (20%–50%) even in inpatient settings [6]. Detailed information from National Health Insurance databases could bring more relevant information about the relationship between amount of financial coverage and number of treated patients and duration of therapy.

Alongside changes in our understanding of motor control theory, the application of methods, such as the Bobath concept, has evolved [3]. Newer methods have also emerged, such as the use of constraint-induced movement therapy, robotics and virtual reality [3,32]. These are based on evidence which has demonstrated that intensive and task-specific practice are key factors for restoration of functions and brain recovery [32]. In light of this, it was anticipated that the majority of respondents might have placed a greater emphasis on employing task oriented interventions which utilized technologies. Contrary with our expectation, our survey results show that PTs across Europe currently report predominantly using hands-on techniques (hands-on may include adaptive resistance, giving reference points in balance training, soft tissue or physical mobilization techniques), followed by verbal instruction (providing advice and information) and demonstration. It is interesting that all European regions, except the North, were similar in this aspect. Real world practice may be lagging behind the recent scientific evidence. One, however, must interpret this with some caution. Whilst exploratory experimental trials can address important questions about what works, they have also often been criticized for being reductionist in their approach compared to the complex interplay of real world settings where presenting problems and solutions are frequently multi-factorial and multi-disciplinary in nature [33]. There is the need for a range of methodologies to be utilized to inform more fully our clinical practice.

A questionnaire survey cannot provide evidence of effectiveness. Whilst it is interesting that the PTs reported that they perceived their intervention is usually effective, with the benefits maintained in the longer term. This perspective, however, is open to bias and hence it is essential that these opinions are verified by well conducted trials, using appropriate methodologies [34]. In this respect, however, it is notable that Western PTs were more sceptical.

Although it has been suggested that patients should attend regular, long-term therapy [8,35], our results indicate that only 43% of PTs are in contact with their patients after the treatment ends. It is possible that this is because patients are receiving PT in the community; although this is not reflected by the survey results. It can also be argued that a contemporary health system has several possibilities how to best support people with long term disabilities through the use of alternative approaches such as the education of patients to self-manage their own condition or the use of electronic health technologies [31]. Telerehabilitation approaches including interactive web-based programs, home-based systems for physical activity monitoring, or online communities [36], for instance, could monitor and motivate patients over time, whilst potentially improving the accessibility and quality of physiotherapy services [31].

Our results also highlighted that only about half of the therapists reported modifying their therapy input according to levels of patient disability. This is not in line with recommendations that needs differ over the disease trajectory and with the level of disability and that intervention should be tailored accordingly [8]. These results are in line with those of Kalron et al. [24] who did not find significant differences in the amount of therapy provision between the mildly and moderately-severely disabled subgroups.

The strengths of this study lie in its relatively high response rate and the representation of respondents from a high number of countries, which allows an extensive description of PT in MS across Europe. However, there are several limitations of the study such as the sample selection and the lengthy nature of the questionnaire [6,7]. Further, the results are based only on the subjective opinion and view of PTs; further research is required to explore this in more depth.

Although there is some consensus on what patients need [1,8,30] and there are recommendations about rehabilitation [9], to date there is limited data to describe what constitutes MS PT practice across Europe. Our survey uncovered variation across European regions in the frequency of using PT interventions, characteristics of therapy sessions, therapy modification and long term monitoring for people with MS. Moreover, our survey results suggest that recommendations concerning treatment adjustment and accessibility are not widely implemented in clinical practice. This knowledge may help support the creation of guidelines for practitioners to improve the consistency and quality of PT delivery, which aim to enhance functioning and quality of life for people with MS.

## 5. Conclusions

The content and delivery of physical therapy differs across Europe. Recommendations concerning access to treatment and adjustment according to disability do not appear to be widely implemented in clinical practice.

## Figures and Tables

**Table 1:** Respondents’ characteristics.

Characteristic	Total		Region								Pearson X^2^-Test *p*-Value
			East		North		South		West		
	N	%	N	%	N	%	N	%	N	%	
Total respondents	212	100.0	35	100.0	65	100.0	91	100.0	21	100.0	
Gender											
Female	154	72.6	25	71.4	56	86.2	63	69.2	10	47.6	0.009 *
Male	58	27.4	10	28.6	9	13.8	28	30.8	11	52.4	
Age											
21–30	65	30.7	17	48.6	15	23.1	25	27.5	8	38.1	0.015 *
31–50	123	58.0	15	42.9	38	58.5	61	67.0	9	42.9	
>50	24	11.3	3	8.6	12	18.5	5	5.5	4	19.0	
Profession #											
Physiotherapist	201	94.8	32	91.4	62	95.4	89	97.8	18	85.7	0.095
Researcher	14	6.6	3	8.6	2	3.1	5	5.5	4	19.0	0.070
Other profession	8	3.8	2	5.7	4	6.2	0	0.0	2	9.5	0.099
Educational level											
Doctoral	18	8.5	6	17.1	2	3.1	10	11.0	0	0.0	<0.001 *
Masters	69	32.5	21	60.0	19	29.2	22	24.2	7	33.3	
Bachelor	87	41.0	6	17.1	39	60.0	31	34.1	11	52.4	
Diploma specialist	23	10.8	0	0.0	3	4.6	19	20.9	1	4.8	
Other education	15	7.1	2	5.7	2	3.1	9	9.9	2	9.5	
Years in Practice											
0–2	28	13.2	12	34.3	8	12.3	6	6.6	2	9.5	<0.001 *
3–10	63	29.7	8	22.9	13	20.0	36	39.6	6	28.6	
>10	121	57.1	15	42.9	44	67.7	49	53.8	13	61.9	
Worktime with MS patients											
0%–24%	87	41.0	22	62.9	18	27.7	40	44.0	7	33.3	0.011 *
25%–49%	40	18.9	4	11.4	10	15.4	22	24.2	4	19.0	
50%–74%	33	15.6	1	2.9	15	23.1	14	15.4	3	14.3	
75%–100%	52	24.5	8	22.9	22	33.8	15	16.5	7	33.3	

* Significant between-region differences (*p* < 0.05); ^#^ Respondents were allowed to report more than one profession.
